# *QuickStats:* Percentage of Children Aged 1–5 Years Who Had Never Been to a Dentist,* by Age and Year — National Health Interview Survey,^^† ^^United States, 2006–2016

**DOI:** 10.15585/mmwr.mm6708a8

**Published:** 2018-03-02

**Figure:**
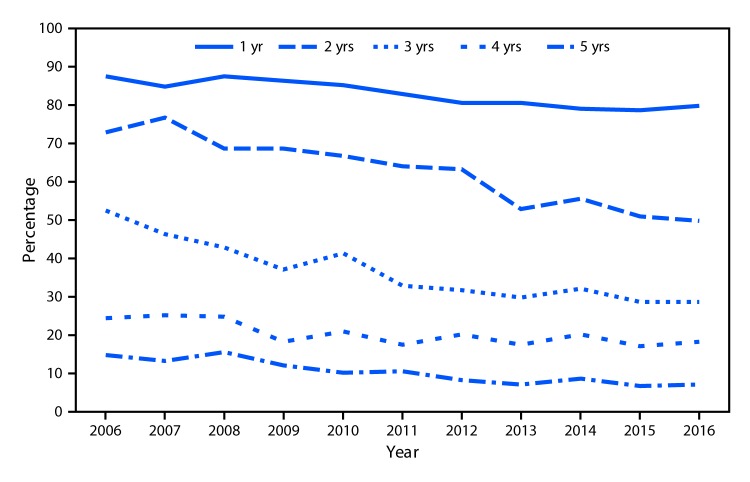
During 2006–2016, the percentage of children aged 1–5 years who had never seen a dentist decreased as age increased. In 2016, 80.2% of children aged 1 year, 49.7% of children aged 2 years, 28.6% of children aged 3 years, 18.3% of children aged 4 years, and 6.8% of children aged 5 years had never seen a dentist. For all ages, the percentage of children who had never seen a dentist declined from 2006 to 2016.

**Source:** National Health Interview Survey, 2006–2016. https://www.cdc.gov/nchs/nhis/index.htm.

